# Machine learning for classification of cutaneous sebaceous neoplasms: implementing decision tree model using cytological and architectural features

**DOI:** 10.1186/s13000-023-01378-w

**Published:** 2023-08-07

**Authors:** Kambiz Kamyab-Hesari, Vahidehsadat Azhari, Ali Ahmadzade, Fahimeh Asadi Amoli, Anahita Najafi, Alireza Hasanzadeh, Alireza Beikmarzehei

**Affiliations:** 1 Department of Dermatopathology, Razi Hospital, Tehran University of Medical Sciences, Tehran, Iran; 2 Medical school, Tehran University of Medical Sciences, Keshavarz Blvd, Tehran, Iran; 3 Department of Pathology, Farabi Eye Hospital, Tehran University of Medical Sciences, Tehran, Iran

**Keywords:** Sebaceous hyperplasia, Sebaceoma, Sebaceous adenoma, Sebaceous carcinoma, Sebaceous neoplasms

## Abstract

**Background:**

This observational study aims to describe and compare histopathological, architectural, and nuclear characteristics of sebaceous lesions and utilized these characteristics to develop a predictive classification approach using machine learning algorithms.

**Methods:**

This cross-sectional study was conducted on Iranian patients with sebaceous tumors from two hospitals between March 2015 and March 2019. Pathology slides were reviewed by two pathologists and the architectural and cytological attributes were recorded. Multiple decision tree models were trained using 5-fold cross validation to determine the most important predictor variables and to develop a simple prediction model.

**Results:**

This study assessed the characteristics of 123 sebaceous tumors. Histopathological findings, including pagetoid appearance, neurovascular invasion, atypical mitosis, extensive necrotic area, poor cell differentiation, and non-lobular tumor growth pattern, as well as nuclear features, including highly irregular nuclear contour, and large nuclear size were exclusively observed in carcinomatous tumors. Among non-carcinomatous lesions, some sebaceoma and sebaceous adenoma cases had features like high mitotic activity, which can be misleading and complicate diagnosis. Based on multiple decision tree models, the five most critical variables for lesion categorization were identified as: basaloid cell count, peripheral basaloid cell layers, tumor margin, nuclear size, and chromatin.

**Conclusions:**

This study implemented a machine learning modeling approach to help optimally categorize sebaceous lesions based on architectural and nuclear features. However, studies of larger sample sizes are needed to ensure the accuracy of our suggested predictive model.

## Background

Sebaceous glands are usually partnered with a hair follicle to form pilosebaceous units, which are widely distributed across the skin. Primarily, these holocrine glands secrete a yellowish, waxy substance called sebum [1]. Few sebaceous glands are also present in hairless regions of skin, such as Meibomian glands in the tarsal region, Fordyce spots in buccal skin, and vermilion of the lip, Montgomery tubules in the areolae, and Tyson glands in prepuce and labia minora [2–4]. The sebaceous glands consist of secretory lobules composed of sebaceous gland cells (sebocytes) and a short tubular squamous duct [5]. At the gland’s outer layer, sebocytes form a layer of undifferentiated germinal cells, which grow toward the center and gradually differentiate into mature sebocytes [6]. As these cells differentiate, their cytoplasm is loaded with lipid vacuoles while other organelles get compressed, and their nucleus gets distorted [5].

There are a limited number of skin lesions with primarily sebaceous origins, namely sebaceous hyperplasia, sebaceous adenoma, sebaceoma, and sebaceous carcinoma [7]. With around one in every four adults, sebaceous hyperplasia is the most prevalent sebaceous lesion; however, it is not commonly considered a true sebaceous neoplasm [7, 8]. Sebaceous adenoma and sebaceoma are benign neoplasms that often develop as yellowish papules on the forehead and cheeks [9]. Sebaceous carcinoma is the only malignant lesion in this list which is commonly divided into periocular and extraocular subtypes and has a rare prevalence of around 0.5 to 2 cases in a million [10, 11]. These lesions can develop independently or be associated with Muir-Torre syndrome [8, 12]. This syndrome is defined by the presence of sebaceous gland tumors or keratoacanthoma that are associated with visceral malignant diseases [13].

Differentiation of benign sebaceous lesions from low-grade malignant tumors has remained a challenge. This observational study aims to describe and compare the architectural, cytological, and histopathological characteristics of sebaceous lesions and use these characteristics to develop a predictive classification approach.

## Methods

### Study design and setting

This is a cross-sectional study conducted on patients with sebaceous neoplasms referred to two hospitals associated with Tehran University of Medical Sciences from March 2015 to March 2019. During the study, pathology slides of sebaceous lesions were retrieved and reviewed. Two independent dermatopathologists classified the retrieved slides according to the established diagnostic criteria, and any possible disagreement was resolved by discussion or consulting a senior pathologist [14, 15]. The study was in concordance with the declaration of Helsinki and its later amendments. The ethical committee of Tehran University of Medical Sciences approved the study (registry code: IR.TUMS.MEDICINE.REC.1399.273).

### Participants

We included patients afflicted with sebaceous neoplasms referred to two university-affiliated hospitals during a five-year period. No age or gender limitations were imposed to include patients. We excluded patients whose data was missing.

### Variables, data sources and measurement

Pathology slides of cases were retrieved from the two hospitals by a pathology resident. Two dermatopathologists classified the retrieved slides. Demographic and histopathologic characteristics of cases were recorded into a checklist for descriptive analysis of each classification of sebaceous lesions. Each slide was assessed regarding architectural and cytological attributes. Architectural attributes included the presence of cellular growth pattern, neural and vascular invasion, circumscribed or infiltrative margins, cystic pattern, necrosis, ductal differentiation, squamous differentiation, ulceration, pagetoid spread to the epidermis, basaloid cell count of more than 50%, and basaloid layers count. Following cytological features were also investigated: degree of cellular differentiation, mitotic activity per 10 high power field, atypical mitosis, nuclear contour, chromatin appearance, and presence of nucleoli. In addition, nuclear size was estimated by comparison with adjacent keratinocyte cells.

### Statistical methods

The collected data was analyzed using Statistical Package for the Social Sciences (SPSS) version 21 (IBM Corp., Armonk, N.Y., USA). Qualitative variables were reported as frequencies and percentages, and quantitative variables were reported as either median and interquartile range (IQR) or mean and standard deviations (SD). Chi-squared tests were used to compare categorical variables between groups. A decision tree method was used to determine the most important predicting variables and develop a prediction model. We used Python’s Scikitlearn library for this approach, and periocular and extraocular carcinomas were considered the same in this analysis. Multiple models were trained using the ExtraTreesClassifier technique, and the five most predictive variables were identified by averaging the feature importance of each variable across all models. To find a single simple and efficient decision tree, multiple decision tree models with depths of 1 up to 10 were cross-validated on our dataset, and the mean accuracy scores were calculated. Accuracy was calculated as the number of correct predictions divided by all predictions. The best decision tree model with a depth of 2 was also identified and visualized using five-fold cross-validation.

## Results

### Participants and descriptive data

A total of 123 cases consisting of 52 sebaceous hyperplasias, 15 sebaceomas, 13 sebaceous adenomas, 20 carcinomas extraocular sebaceous, and 23 periocular sebaceous carcinomas were identified to be included in the study. 65.9% of patients were male, and the median age was 63.5 (IQR 20). No significant difference was observed between the mean age of male (62.7, SD 16.5) and female (59.3, SD 19.0) patients (P value 0.33). However, a comparison of mean age between patients with extra- or periocular sebaceous carcinomas (68.8, SD 16.4) and those with benign lesions (56.87, SD 16.5) showed a significant difference (P value 0.00). Age and gender distribution for each lesion are summarized in Table 1. The details of each tumor’s histopathological and nuclear characteristics are presented in Tables 2 and 3. Comparison of characteristics between combined cases of extra- and periocular sebaceous carcinomas and each benign lesion and between sebaceous adenomas and other benign lesions are summarized in Table 4. Here we briefly discuss each lesion.

**Table 1:** Demographic characteristics of different sebaceous lesions

Lesion type	Male no. (%)	Female no. (%)	Median age (IQR)
Sebaceous hyperplasia	32 (61.5%)	20 (38.5%)	57.0 (24)
Sebaceoma	12 (80.0%)	3 (20.0%)	64.5 (21)
Sebaceous adenoma	11 (84.6%)	2 (15.4%)	69.0 (24)
Extraocular sebaceous carcinoma	16 (80.0%)	4 (20.0%)	67.5 (33)
Periocular sebaceous carcinoma	10 (43.5%)	13 (56.5%)	72.0 (23)

**Table 2:** Histopathological characteristics of different sebaceous lesions

Variables	Sebaceous hyperplasia (n = 52)	Sebaceoma (n = 15)	Sebaceous adenoma (n = 13)	Extraocular sebaceous carcinoma (n = 20)	Periocular sebaceous carcinoma (n = 23)
Pagetoid appearance	0 (0.0%)	0 (0.0%)	0 (0.0%)	3 (15.0%)	10 (43.4%)
Vascular invasion	0 (0.0%)	0 (0.0%)	0 (0.0%)	1 (5.0%)	3 (13.0%)
Neural invasion	0 (0.0%)	0 (0.0%)	0 (0.0%)	1 (5.0%)	4 (17.3%)
Atypical mitosis	0 (0.0%)	0 (0.0%)	0 (0.0%)	7 (35.0%)	17 (73.9%)
Cystic appearance	0 (0.0%)	5 (33.3%)	4 (30.8%)	4 (20.0%)	7 (30.4%)
Ulceration and erosion	0 (0.0%)	5 (33.3%)	7 (53.8%)	15 (75.0%)	18 (78.2%)
Ductal differentiation	0 (0.0%)	12 (80.0%)	2 (15.4%)	16 (80.0%)	5 (21.7%)
Squamous differentiation	0 (0.0%)	7 (46.7%)	1 (7.7%)	11 (55.0%)	10 (43.5%)
Basaloid cell count					
< 50%	52 (100.0%)	0 (0.0%)	13 (100.0%)	1 (5.0%)	0 (0.0%)
> 50%	0 (0.0%)	15 (100.0%)	0 (0.0%)	19 (95.0%)	23 (100.0%)
Tumor margin					
Circumscribed	52 (100.0%)	15 (100.0%)	13 (100.0%)	3 (15.0%)	4 (17.4%)
Infiltrative	0 (0.0%)	0 (0.0%)	0 (0.0%)	17 (85.0%)	19 (82.6%)
Necrotic area					
None	52 (100.0%)	14 (93.3%)	13(100.0%)	7 (35.0%)	10 (43.5%)
Single cell	0 (0.0%)	1 (6.7%)	0 (0.0%)	0 (0.0%)	0 (0.0%)
Narrow	0 (0.0%)	0 (0.0%)	0 (0.0%)	7 (35.0%)	9 (39.1%)
Extensive	0 (0.0%)	0 (0.0%)	0 (0.0%)	6 (30.0%)	4 (17.4%)
Cell differentiation					
Well	52 (100.0%)	6 (40.0%)	12 (92.3%)	0 (0.0%)	4 (17.4%)
Moderate	0 (0.0%)	9 (60.0%)	1 (7.7%)	9 (45.0%)	13 (56.5%)
Poor	0 (0.0%)	0 (0.0%)	0 (0.0%)	11 (55.0%)	6 (26.1%)
Tumor growth pattern					
Lobular	52 (100.0%)	15 (100.0%)	13 (100.0%)	10 (50.0%)	11 (47.8%)
Comedo	0 (0.0%)	0 (0.0%)	0 (0.0%)	6 (30.0%)	4 (17.4%)
Papillary	0 (0.0%)	0 (0.0%)	0 (0.0%)	0 (0.0%)	0 (0.0%)
Mixed	0 (0.0%)	0 (0.0%)	0 (0.0%)	4 (20.0%)	8 (34.8%)
Peripheral basaloid layers					
≤ 2	52 (100.0%)	0 (0.0%)	9 (69.2%)	1 (5.0%)	0 (0.0%)
> 2	0 (0.0%)	15 (100.0%)	4 (30.8%)	19 (95.0%)	23 (100.0%)
Mitotic activity (per 10 HPF)					
0–1	52 (100.0%)	6 (40.0%)	10 (76.9%)	1 (5.0%)	1 (4.3%)
2–5	0 (0.0%)	6 (40.0%)	2 (15.4%)	2 (10.0%)	4 (17.4%)
> 5	0 (0.0%)	3 (20.0%)	1 (7.7%)	17 (85.0%)	18 (78.3%)

**Table 3:** Nuclear characteristics of different sebaceous lesions

Variables	Sebaceous hyperplasia (n = 52)	Sebaceoma (n = 15)	Sebaceous adenoma (n = 13)	Extraocular sebaceous carcinoma (n = 20)	Periocular sebaceous carcinoma (n = 23)
Chromatin					
Fine	52 (100.0%)	8 (53.3%)	5 (38.5%)	0 (0.0%)	1 (4.3%)
Coarse	0 (0.0%)	4 (26.7%)	3 (23.1%)	5 (25.0%)	3 (13.0%)
Clumpy	0 (0.0%)	2 (13.3%)	4 (30.8%)	3 (15.0%)	1 (4.3%)
Vesicular	0 (0.0%)	1 (6.7%)	1 (7.7%)	12 (60.0%)	12 (52.2%)
Mixed	0 (0.0%)	0 (0.0%)	0 (0.0%)	0 (0.0%)	6 (26.1%)
Nucleoli					
Inconspicuous	48 (92.3%)	2 (13.3%)	2 (15.4%)	1 (5.0%)	3 (13.0%)
One evident	4 (7.7%)	5 (33.3%)	4 (30.8%)	2 (10.0%)	6 (26.1%)
One prominent	0 (0.0%)	5 (33.3%)	3 (23.1%)	7 (35.0%)	6 (26.1%)
Multiple	0 (0.0%)	3 (20.0%)	4 (30.8%)	10 (50.0%)	8 (34.8%)
Nuclear contour					
Smooth	52 (100.0%)	13 (86.7%)	13 (100.0%)	4 (20.0%)	0 (0.0%)
Irregular mild	0 (0.0%)	2 (13.3%)	0 (0.0%)	5 (25.0%)	4 (17.4%)
Irregular moderate	0 (0.0%)	0 (0.0%)	0 (0.0%)	7 (35.0%)	12 (52.2%)
Significant	0 (0.0%)	0 (0.0%)	0 (0.0%)	4 (20.0%)	7 (30.4%)
Nuclear size (relative to nuclear size adjacent keratinocytes)					
Less than 1 times	52 (100.0%)	10 (66.7%)	7 (53.8%)	1 (5.0%)	0 (0.0%)
Between 1 and 2 times	0 (0.0%)	5 (33.7%)	6 (46.2%)	9 (45.0%)	4 (17.4%)
More than 2 times	0 (0.0%)	0 (0.0%)	0 (0.0%)	10 (50.0%)	19 (82.6%)

**Table 4:** Comparison of histological characteristics between carcinomatous tumors and each benign lesion and between sebaceous adenoma and other benign lesions (P values)

Characteristics	SH vs. CT	SB vs. CT	SA vs. CT	SH vs. SA	SB vs. SA
Pagetoid appearance	0.00	0.02	0.05	-	-
Vascular invasion	0.03	0.56	0.56	-	-
Neural invasion	0.01	0.30	0.31	-	-
Atypical mitosis	0.00	0.00	0.00	-	-
Cystic appearance	0.00	0.74	1	0.00	1
Ulceration and erosion	0.00	0.01	0.17	0.00	0.34
Ductal differentiation	0.00	0.03	0.03	0.03	0.00
Squamous differentiation	0.00	0.88	0.00	0.20	0.03
Basaloid cell count	0.00	1	0.00	-	0.00
Tumor margin	0.00	0.00	0.00	-	-
Necrotic area	0.00	0.00	0.00	-	1
Cell differentiation	0.00	0.00	0.00	0.20	0.00
Tumor growth pattern	0.00	0.00	0.00	-	-
Peripheral basaloid layers	0.00	1	0.00	0.00	0.00
Mitotic activity	0.00	0.00	0.00	0.00	0.14
Chromatin	0.00	0.00	0.00	0.00	0.71
Nucleoli	0.00	0.47	0.70	0.00	0.89
Nuclear contour	0.00	0.00	0.00	-	0.48
Nuclear size	0.00	0.00	0.00	0.00	0.48

SH: Sebaceous Hyperplasia, CT: Carcinomatous tumors, SB: Sebaceoma, SA: Sebaceous adenoma

## Main results

### Sebaceous hyperplasia

**Fig. 1:**
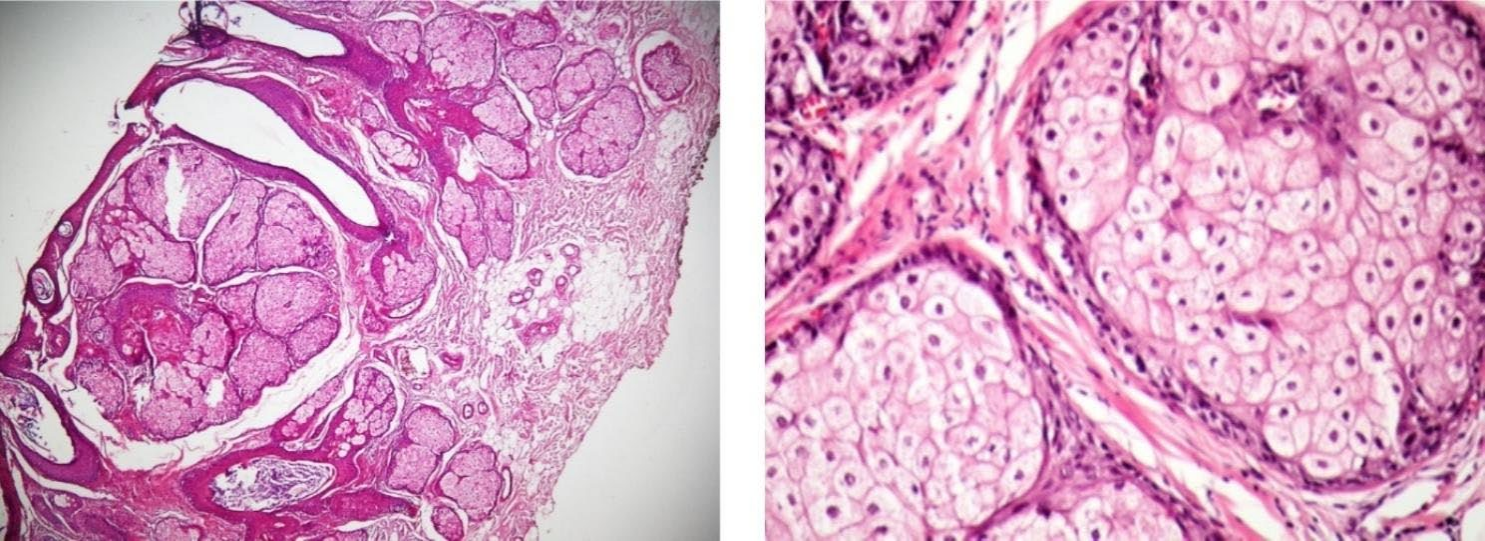
**A**, Sebaceous hyperplasia reveals well-demarcated sebaceous lobules in low power magnificent. **B**, Hyperplastic sebaceous lobules reflect the normal sebaceous gland and consist of a maximum of two outer layers of basaloid cells surrounding mature sebaceous cells with eosinophilic bubbly cytoplasm

No atypical appearance, neurovascular invasion, atypical mitosis, necrotic area, or ulceration was observed. All cells were well-differentiated and circumscribed, and the number of peripheral basaloid layers did not exceed two (Fig. 1A). The nuclear contour was smooth, chromatin was fine, and nucleoli were inconspicuous in most cells. Nucleus sizes were less than that of adjacent keratinocytes (Fig. 1B).

### Sebaceoma

**Fig. 2:**
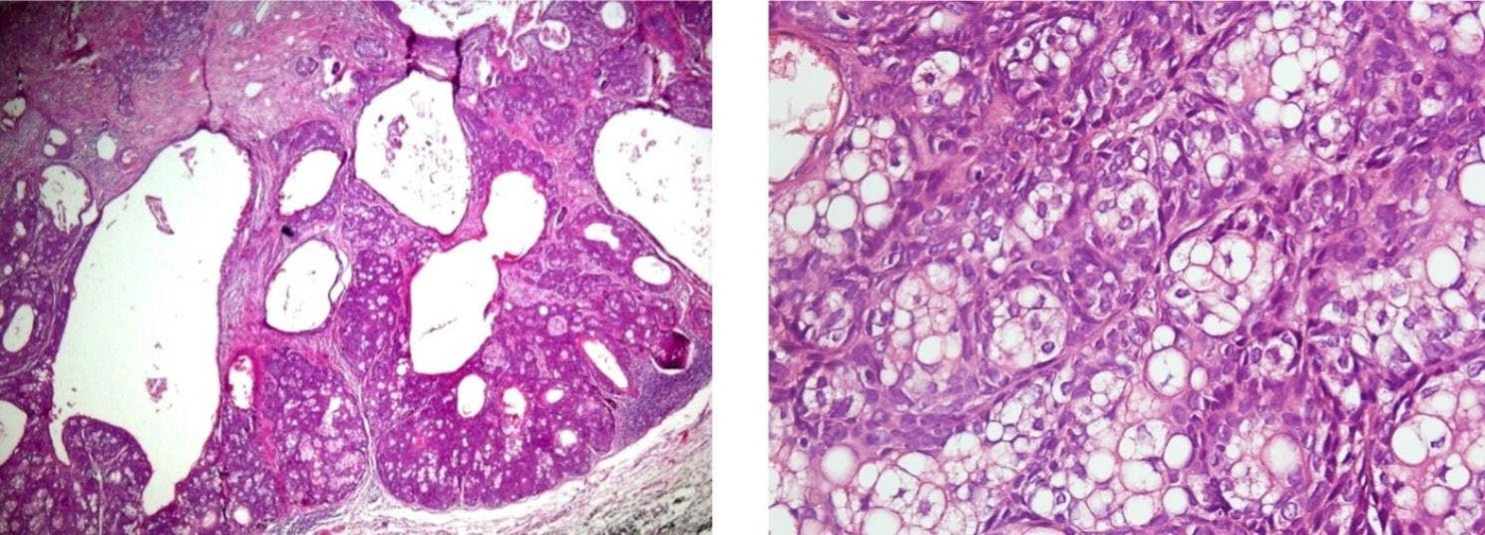
**A**, Sebaceomas are well- circumscribed with conspicuous cyst formation. **B**, Mature sebocytes are mixed with basaloid cells in a high-power view

Cystic appearance and ulceration were present in some, and squamous and ductal differentiation was observed in more than half of the cases (Fig. 2A). The majority of the cells were moderately differentiated and circumscribed, and more than two peripheral basaloid layers were seen in all of these cases. The nuclear contour was mostly smooth, and at least one nucleolus was present in most cells (Fig. 2B). Chromatin was fine or coarse in most cases, and nucleus size was less than twice of keratinocytes.

### Sebaceous adenoma

**Fig. 3:**
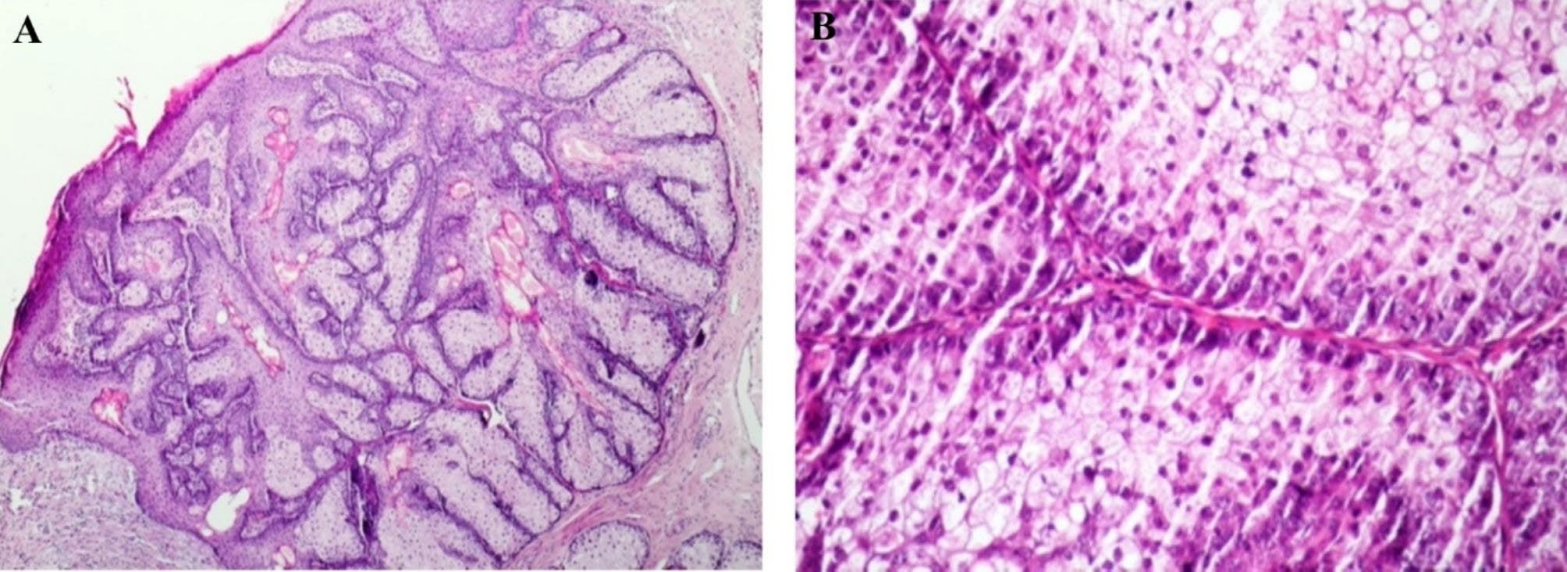
**A**, Sebaceous adenoma with sharply circumscribed sebaceous lobules contiguous with the epidermis, surrounded by a compressed pseudo capsule of dermal stroma. **B**, Higher power of a sebaceous adenoma reveals an expansion of germinative basaloid cell layers at periphery, germinative cells populace, with centrally located mature sebaceous cyst

Ulceration was observed in half of the cases, and some cases demonstrated cystic appearance and ductal differentiation. Most cases were well-differentiated and circumscribed without any necrotic regions (Fig. 3A). A variable number of peripheral basaloid cell layers was observed. No nuclear contour irregularity was seen in any of the cases; however, coarse and clumpy chromatin and prominent nucleoli were observed in several cases. Nucleus sizes were less than twice those of keratinocytes (Fig. 3B).

### Extraocular sebaceous carcinoma

**Fig. 4:**
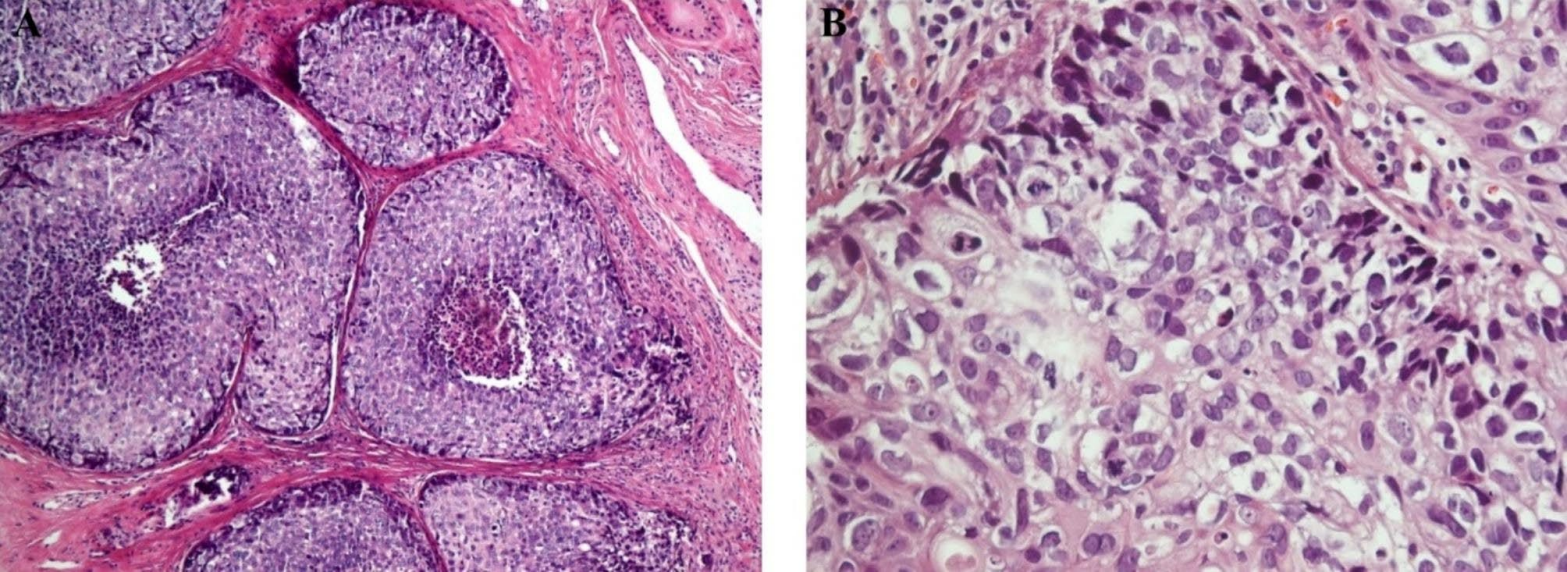
**A**, Poorly differentiated extraocular sebaceous carcinoma with comedo necrosis. **B**, Tumoral cells show scant cytoplasmic vacuolation, marked atypical mitoses and nuclear polymorphism in high magnificent

Ductal differentiation and ulceration were prominently present, and atypical mitosis and necrotic areas were observed in several cases (Fig. 4A). Tumor margins were mostly infiltrative, and no evidence of well differentiation was observed in any of the cases. Most cases had more than two layers of peripheral basaloid cells. Vesicular chromatin was present in more than half of the cases, and nuclear contour irregularity was prevalent. Most cells had at least one prominent nucleolus, and their nucleus sizes were greater than keratinocytes (Fig. 4B).

### Periocular sebaceous carcinoma

**Fig. 5:**
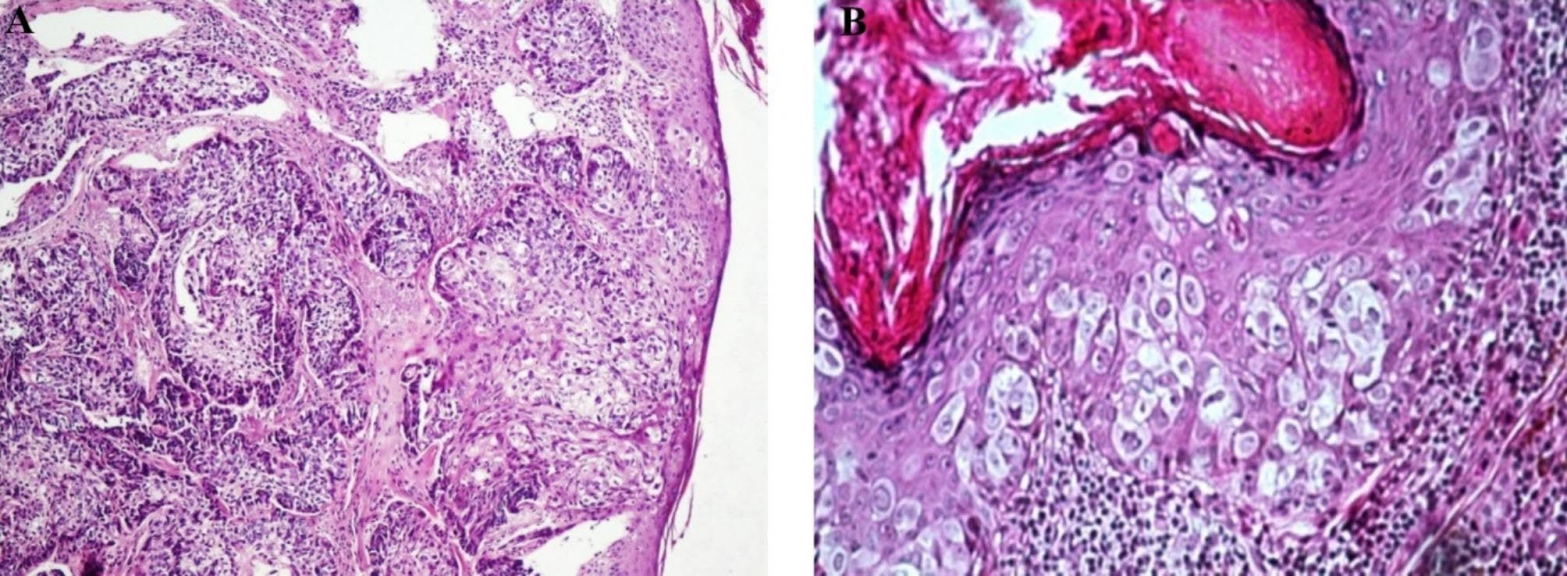
**A**, Periocular sebaceous carcinoma with infiltrative pattern of tumoral cells in the eye lid desmoplastic stroma. **B**, Pagetoid invasion of sebaceous gland carcinoma in the epidermis of eyelid

Atypical mitosis and ulceration were prevalently observed, and squamous differentiation and cystic and pagetoid appearances were seen in many cases (Fig. 5B). The majority of cases had infiltrative margins and moderate/poor cell differentiation (Fig. 5A). More than two peripheral basaloid cell layers were present in all cases. Nucleoli were observed in most of the cells.

### Prediction model

**Fig. 6:**
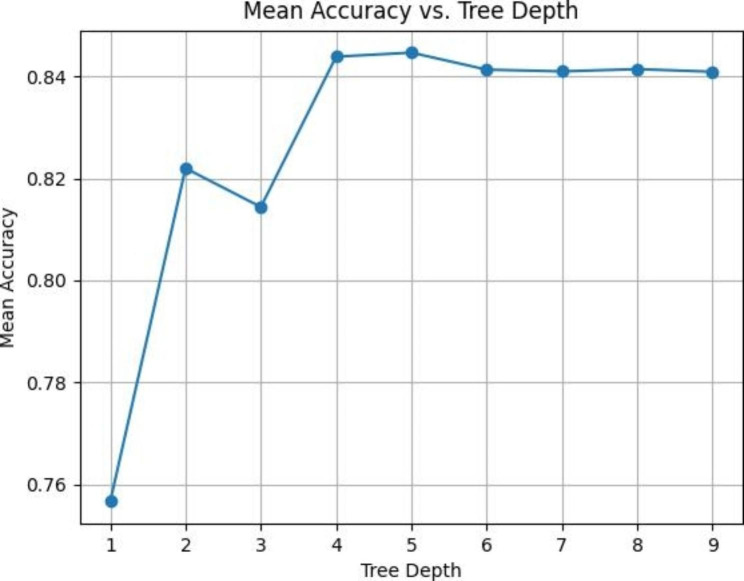
The mean accuracy of multiple decision tree models with depths ranging from 1 to 10

**Fig. 7:**
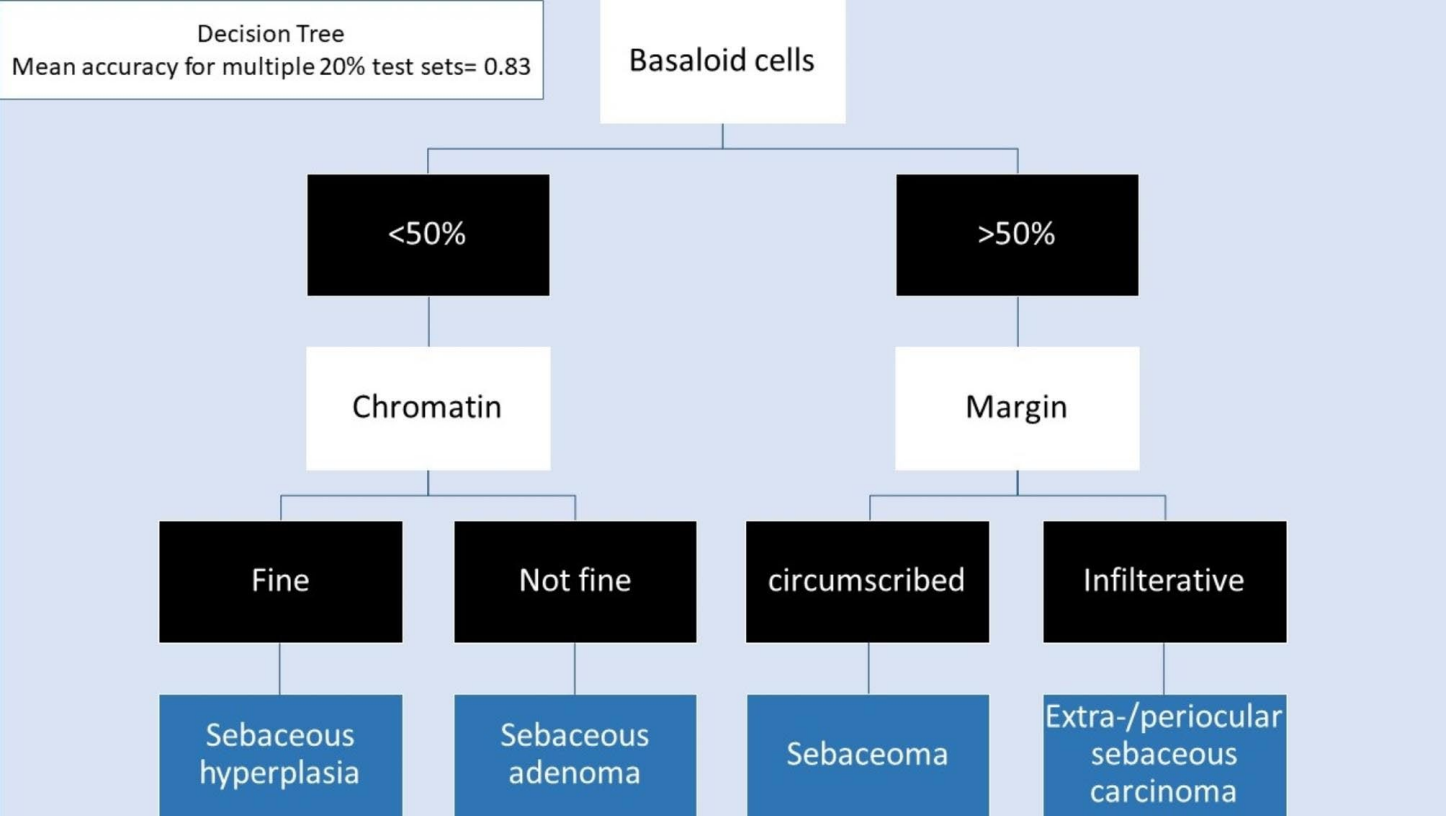
The most accurate decision tree prediction model with a depth of 2

The five most important predictive variables included: basaloid cell count, peripheral basaloid cell layers, tumor margin, nuclear size, and chromatin. The mean accuracy after cross-validation for multiple models with depths ranging from 1 to 10 is summarized in Fig. 6. Details of the best prediction model with a depth of 2 are depicted in Fig. 7.

## Discussion

This study describes and compares detailed nuclear, cytological, and architectural characteristics of sebaceous tumors and highlights the distinctive nuclear features of sebaceous neoplasms.

There is a debate on the distinction of sebaceous adenomas from carcinomas regarding current evidence. The currently available grading criteria are predominantly based on architectural features [7]. Only a fair to moderate degree of interobserver agreement among the specialized dermatopathologists has been reported [16]. While some authors have proposed that sebaceous adenomas should be considered malignant due to mitotic features, nuclear crowding, disorganized arrangement of mature and immature cells, and pleomorphism [17–20], the majority of experts categorize them as benign lesions [21]. Moreover, the distinction of sebaceoma from sebaceous carcinoma is generally based on invasive growth and pleomorphism of the latter. However, similar to the issue with sebaceous adenomas, the classification of a minor group of lesions that show well circumscription with a discordant degree of atypia remains controversial [22]. Our study suggests that the role of nuclear features should be taken into account for a more accurate diagnosis of cutaneous sebaceous neoplasm. The most predominant pleomorphic features observed in our study were the enlarged nuclear size, prominent nucleoli, and coarse chromatin present in sebaceomas, sebaceous adenomas, and carcinomas. Multiple nucleoli were primarily observed in malignant carcinomas, but intergroup differences with benign lesions did not reach a level of significance. Nuclear contour irregularity was only observed in carcinomas and two cases of sebaceoma. Mitotic activity was observed in both benign lesions and carcinomas, but there was a significant difference in favor of higher mitotic activity in malignant cases. Nucleolar and chromatin features between sebaceous adenoma and sebaceoma were statistically closer to sebaceous carcinomas than sebaceous hyperplasia. A statistical similarity was observed between sebaceoma and sebaceous carcinomas regarding the basaloid cell count. This finding can be explained by the current definition of sebaceoma, which indicates a germinative cell count of more than 50% as a cut-off value for distinguishing sebaceoma from sebaceous adenoma [7]. Ulceration and erosion were seen in nearly half of our cases and reached a significant level. Ulceration has previously been reported in sebaceous adenoma. However, some authors have suggested that it should be considered as a feature of malignancy that prompts careful assessment [17, 19].

Enlarged nuclear and nucleolar sizes, hyperchromasia, mitotic figures, decreased differentiation, and necrosis has been previously reported in malignant sebaceous tumors. Our findings regarding the nuclear contour in sebaceous adenoma and sebaceoma were consistent with the previous studies. However, in contrast to the previous findings, we found coarse chromatin in an increasing number of sebaceoma and sebaceous adenoma cases [8, 19, 21–23]. The observed pleomorphic features in both benign and malignant lesions of sebaceous glands, the relatively high interobserver variability [16], and the necessity of accurate diagnosis underline the importance of utilizing more reliable criteria to avoid mismanagements and tumor recurrences.

### Decision tree algorithm

We proposed a machine-learning-based predictive modeling approach to obtain a descriptive model that classifies cases based on decision rules inferred from the features of a given set of studied variables. Decision trees are powerful classification tools that are easy to interpret and visualize and can handle problems with multiple outputs. However, they must be applied carefully since minor variations in the data might lead to a different classification algorithm. Additionally, a careful choice of parameters in the applied algorithm is necessary to avoid an over-complex model and increase the model’s generalizability. The decision tree algorithm has been implemented in previous studies as well [24–27]. In our study, the main predictive variables were peripheral basaloid cell layers and count, chromatin characteristics, nuclear size, and tumor margin. Our suggested decision tree model is highly consistent with a recent study that implemented this method to classify sebaceous neoplasms. Nevertheless, we cross-validated our model on five different folds of the dataset to reduce overfitting and mitigate the effects of chance associated with fitting on a single random data splitting [28]. Details of our most accurate model, with an accuracy of 83%, are shown in Fig. 7.

### Limitations

Several limitations of this study require consideration. The small sample size of our study undermines the generalizability of our model. We applied model training with different random train and test subsets of our data and reported the mean obtained accuracy. The possibility of misclassification of the specimens cannot be ruled out due to relatively high interobserver variation in the diagnosis of sebaceous lesions. Two independent specialized dermatopathologists, with guidance from a senior pathologist, reviewed the slides to reduce the interobserver variation effect. Another strength of our study lies in utilizing cross-validation to reduce the overfitting of the model.

## Conclusions

The currently used classification criteria of sebaceous neoplasms rely mostly on architectural features and contain many diagnostic gray areas in cases of well-circumscribed architecture. This issue has led to high variability in diagnosis. We described these lesions’ cytological and nuclear features in a more detailed manner. We also implemented a novel modeling approach to help distinguish well-circumscribed lesions more easily. Studies of larger sample sizes are needed to ensure the accuracy of our suggested predictive model. Moreover, understanding the biological basis of these lesions may allow for a better concordant classification system.

## Data Availability

The datasets used and/or analysed during the current study are available from the corresponding author on reasonable request.
